# Association of plasma osteoprotegerin levels with the severity of lower extremity arterial disease in patients with type 2 diabetes

**DOI:** 10.1186/s12872-015-0079-0

**Published:** 2015-08-11

**Authors:** Yixin Niu, Weiwei Zhang, Zhen Yang, Xiaoyong Li, Jie Wen, Suijun Wang, Hongmei Zhang, Xuanchun Wang, Houguang Zhou, Wenjun Fang, Li Qin, Qing Su

**Affiliations:** Department of Endocrinology, Xinhua Hospital Affiliated to Shanghai Jiaotong University School of Medicine, 1665 Kongjiang Road, Shanghai, China; Department of Endocrinology, Huashan Hospital Affiliated to Fudan University, Shanghai, China; Department of Endocrinology, Clinical Geriatric Medicine, Henan Provincial People’s Hospital, Zhengzhou, China; Department of Geriatrics, Huashan Hospital Affiliated to Fudan University, Shanghai, China

## Abstract

**Background:**

Osteoprotegerin (OPG) is a member of the tumor necrosis factor receptor superfamily and suggested as a marker of atherosclerosis. However, little is known about the association between plasma OPG levels and lower extremity arterial disease. We investigated whether plasma OPG levels were associated with the presence and severity of lower extremity arterial disease in patients with type 2 diabetes.

**Methods:**

This was a study of 712 patients with type 2 diabetes aged 40 years or older. Plasma OPG was measured using ELISA. The lower extremity arterial disease was diagnosed by high-frequency color Doppler ultrasonic.

**Results:**

Of 712 patients, 505 (70.9 %) had lower extremity arterial stenosis. OPG levels were significantly increased in patients with lower extremity arterial stenosis [1.89 (1.48-2.41) vs. 2.39 (1.82-3.33) ng/mL, *p* < 0.001]. Plasma OPG levels increased gradually with increasing severity of lower extremity arterial stenosis (*p* < 0.001 for trend), after adjustment for traditional cardiovascular risk factors such as age, gender, smoking, total cholesterol, high-density lipoprotein (HDL) cholesterol, C-reactive protein (CRP), body mass index (BMI), systolic blood pressure(SBP). The risk of lower extremity arterial disease was increased (OR = 1.17, 95 % CI 1.09 –1.28, *p* < 0.001) with each standard deviation (SD) higher level of OPG in patients with type 2 diabetes after adjustment for traditional CVD risk factors.

**Conclusions:**

Plasma OPG levels were significantly associated with the presence and severity of lower extremity arterial disease. Our results suggest that OPG is an important plasma biomarker of lower extremity arterial disease in type 2 diabetes.

## Backgrounds

Osteoprotegerin (OPG) is a soluble member of the TNF–receptor superfamily and act as decoy receptor for both the receptor activator of nuclear factor-B ligand (RANK–L) and the TNF–related apoptosis inducing ligand (TRAIL), two cytokines of the TNF–family [1]. OPG is produced by a variety of tissues, including the cardiovascular system, lung, kidney, and immune tissues, as well as bone, and that the expression and production of OPG are regulated by various cytokines and hormones [2].

Recent studies have indicated that OPG also acts as an important regulatory molecule in vascular disease, such as arterial calcification and atherosclerosis [3–5]. The function of OPG in the arterial wall is not known, but it has been suggested that the molecule acts as a vascular calcification inhibitor due to the fact that OPG knock-out mice develop arterial calcifications [6]. Atherosclerosis is now understood to be an inflammatory disease, and not merely the result of the passive accumulation of lipids within arterial walls [7]. OPG is expressed in vascular cells such as coronary smooth muscle cells and endothelial cells in vitro. In endothelial cells, OPG has been demonstrated to act as an anti-apoptotic factor. Moreover, OPG immunoreactivity was demonstrated not only in the nondiseased vessel wall, but also in early atherosclerotic lesions in human tissues. These findings suggested that OPG may play a pivotal role in the development of vascular disease. A clinical study reported that there was a significant correlation between elevated serum OPG levels and cardiovascular mortality, suggesting that OPG may contribute to the progression of coronary artery disease (CAD), and that they were increased in elderly people and in diabetic patients [8].

Several studies have suggested that circulating OPG levels is positively associated with peripheral arterial disease [9, 10], which defined using ankle brachial index (ABI), although data is inconsistent in recent meta analysis [11]. However, little is known about the association between plasma OPG levels and lower extremity arterial disease, which diagnosed by ultrasonic evaluation, in patients with type 2 diabetes. In this study, we investigated whether plasma OPG levels were associated with the presence and severity of lower extremity arterial disease in patients with type 2 diabetes.

## Methods

### Patients

Subjects were recruited from the Department of Endocrinology at Xinhua Hospital Affiliated to Shanghai Jiaotong University between 2013 and 2014. All unrelated subjects with T2DM who attended the Diabetes Clinic at the Xinhua Hospital were recruited consecutively to participate in a prospective study to identify the risk factors predisposing to the development of diabetic complications. Each visit comprised clinical assessments and laboratory investigations to determine the control of diabetes and related cardiovascular risk factors, and the presence of diabetic complications. Diabetes was defined according to the 2008 American Diabetes Association diagnostic criteria (MM) [12]. Subjects with arteriovenous grafts/shunts,vasculitis, chronic kidney disease, cerebral infarction, coronary artery disease, malignancies, or with immunological diseases, osteoporosis and subjects receiving systemic glucocorticoids or immunosuppressants were excluded from the study. A total of 712 T2DM subjects, who attended regular visits at least twice a year, with the latest follow-up in or before October 2014, were enrolled in the study. Of these, 505 patients with lower extremity arterial stenosis and 207 patients without lower extremity arterial stenosis. Written informed consent was obtained from all the participants. The study was approved by the Institutional Review Broad of Xinhua Hospital Affiliated to Shanghai Jiaotong University School of Medicine.

### Biochemical measurements and clinical data collection

Peripheral venous blood samples were collected after an overnight fast. The fasting plasma glucose(FPG) was measured with the use of the glucose oxidase method on an auto-analyzer (Modular P800; Roche, Basel, Switzerland). Triglycerides, total cholesterol, LDL-cholesterol, HDL-cholesterol, and serum uric acid were measured using chemiluminescence methods on the auto-analyzer (Modular E170; Roche). HbA1c was measured with the use of the Chromatography method on an auto-analyzer (D10; Bio-Rad, USA). Age and history of cigarette use were assessed through an interview preceding the physical examination. We defined “smoking” as the current smokers, who smoke at least one cigarette per day.

Anthropometric parameters were measured in all subjects. Hypertension was defined by systolic blood pressure (SBP) ≥ 140 mmHg, diastolic blood pressure (DBP) ≥ 90 mmHg, the current use of antihypertensive treatment, or a combination of the three. Three blood pressure recordings were obtained from the right arm of patients in a sitting position after 30 min of rest; measurements were taken in 5-min intervals, and mean values were calculated. Body mass index (BMI) was calculated as body weight × height^−2^ and expressed in kg/m^2^. The homeostasis model assessment of insulin resistance (HOMA–IR) was calculated according to the equation described by Matthews et al. [13] The waist circumference (WC) was measured at the smallest circumference between the rib cage and the iliac crest, with the subject in the standing position.

### Measurement of Osteoprotegerin, CRP and Adiponectin

Plasma samples were isolated from fasting subjects and stored at -80 °C prior to analysis. The plasma OPG, C–reactive protein(CRP) and adiponectin were determined in duplicate by ELISA with Duoset kit (DY805, DY1707, and DY1065; R&D Systems, Minneapolis, MN) as recommended by the manufacturer. The ELISA system had an intra-assay coefficient of variation of 3–9 % and an inter-assay coefficient of variation of 4–10 %, respectively.

### Determination of lower extremity arterial disease

The lower extremity arterial stenosis was characterized using high-frequency color Doppler ultrasonic evaluation (Philips, iU22, USA). Based on the criteria established by the 2006 American Society of Echocardiography and the Society of Vascular Medicine and Biology [14], the degree of peripheral arterial diameter reduction was classified as “normal”(0 % diameter reduction of lower extremity arterial stenosis, “mild” (1 –19 %), “moderate” (20 –49 %), or “severe” (50 –99 %). Current and past medical history, personal background, and medication were recorded for all subjects.

### Statistical analysis

The distribution of data was tested by the Kolmogorov-Smirnov test. Normally distributed data were expressed as means ± SD, whereas variables with a skewed distribution were reported as median (inter-quartile range) and log transformed to approximate normality before analysis. Differences between groups were tested using the unpaired Student’s *t* test or *χ*2 analysis, as appropriate. Pearson correlations were computed to assess the relationship between variables and OPG. Plasma OPG levels were depicted according to the severity of lower extremity arterial disease using linear regression model. To investigate the association of the lower extremity arterial disease with OPG, we defined participants with normal lower extremity artery as 0 (*n* = 207) and the lower extremity arterial disease as 1 (*n* = 505) in the logistic regression analyses. Potential confounding variables including age, gender and current smoking, BMI, waist/hip ratio and HOMA-IR, SBP, DBP, CRP, HbA1c, Triglycerides, total cholesterol, HDL-C and LDL-C were controlled in the regression models. Data management and statistical analysis were performed with the SPSS Statistical Package (version 13.0; SPSS Inc., Chicago, IL). *P* values < 0.05 was considered statistically significant.

## Results

### Baseline characteristics

There were 505 (70.9 %) patients with lower extremity arterial stenosis and 207 (29.1 %) patients without lower extremity arterial stenosis. Clinical, biochemical and metabolic characteristics of participants of the study were given in Table 1.

**Table 1:** Characteristics of study population with or without lower extremity arterial stenosis

	Without lower extremity arterial stenosis	Lower extremity arterial stenosis	*p* value
n	207	505	-
Age (years)	53.5 ± 12.1	65.3 ± 11.0	<0.001
Gender (male/female)	308/197	112/95	0.094
Duration of diabetes (years)	6.3 ± 5.9	10.6 ± 8.1	<0.001
smoking (%)	50 (24.3 %)	145 (28.7 %)	0.230
Hypertension (%)	86 (41.5 %)	326 (64.6 %)	<0.001
BMI (kg/m2)	25.1 ± 4.2	24.6 ± 3.4	0.184
WC (cm)	94.2 ± 13.1	91.9 ± 11.3	0.106
SBP (mmHg)	130.2 ± 15.1	132.4 ± 15.4	0.199
DBP (mmHg)	78.6 ± 11.5	76.2 ± 9.5	0.038
FPG (mmol/l)	8.5 ± 3.3	8.6 ± 3.2	0.719
HbA1c (%)	9.7 ± 2.5	9.5 ± 2.3	0.490
(mmol/mol)	83 ± 19.8	80 ± 18.2	0.490
Serum uric acid (mmol/l)	300.3 ± 90.7	303.9 ± 89.0	0.724
Total cholesterol (mmol/l)	4.5 ± 1.1	4.7 ± 1.1	0.091
triglycerides (mmol/l)	1.54 (1.04–2.23)	1.55 (1.03–2.25)	0.962
LDL-c (mmol/l)	2.7 ± 0.8	2.9 ± 0.7	0.122
HDL-c (mmol/l)	1.4 ± 0.3	1.3 ± 0.3	0.203
Adiponectin (mg/l)	9.2 ± 5.7	6.7 ± 4.4	<0.001
CRP (mg/l)	1.75 (0.77–5.38)	2.43 (0.81–5.47)	<0.001
Osteoprotegerin (ng/ml)	1.89 (1.48–2.41)	2.39 (1.82–3.33)	<0.001
Hypoglycemic treatments			0.001
Insulin (%)	78	188	
OHA (%)	100	186	
Insulin + OHA (%)	29	131	

Data are means ± SD, medians (interquartile range), or n (%). P values were obtained by an unpaired Student *t* test or *χ*2 analysis, as appropriate. *BMI* body mass index, *WC* waist circumference, *SBP* systolic blood pressure, *DBP* diastolic blood pressure, *FPG* fasting plasma glucose, *LDL-c* low-density lipoprotein cholesterol, *HDL-c* high-density lipoprotein cholesterol, *OHA* oral hypoglycemic agent

The plasma OPG levels were significantly increased in patients with lower extremity arterial stenosis [1.89 (1.48-2.41) vs. 2.39 (1.82-3.33) ng/mL, *p* < 0.001]. Plasma OPG levels correlated positively with age(r = 0.410, *p* < 0.001), duration of diabetes(r = 0.307, *p* < 0.001), SBP(r = 0.207, *p* < 0.001) and CRP(r = 0.247, *p* < 0.001). Plasma OPG levels were not associated with BMI, WC, DBP, FPG, HbA1C, uric acid, total cholesterol, triglycerides, LDL-C, HDL-C and adiponectin (Table 2).

**Table 2:** Correlation between plasma osteoprotegerin (OPG) levels and biochemical parameters

	r	*p* value
Age	0.410	<0.001
Duration of diabetes	0.307	<0.001
BMI	0.024	0.629
WC	0.030	0.553
SBP	0.207	<0.001
DBP	0.015	0.723
FPG	0.111	0.011
HbA1c (%)	0.020	0.680
Serum uric acid	0.005	0.906
Total cholesterol	0.03	0.521
triglycerides	0.003	0.953
LDL-c	0.036	0.423
HDL-c	0.012	0.792
Adiponectin	−0.03	0.518
CRP	0.247	<0.001

*BMI* body mass index, *WC* waist circumference, *SBP* systolic blood pressure, *DBP* diastolic blood pressure, *FPG* fasting plasma glucose, *LDL-c* low-density lipoprotein cholesterol, *HDL-c* high-density lipoprotein cholesterol

### Association between OPG and severity of lower extremity arterial stenosis

**Fig. 1:**
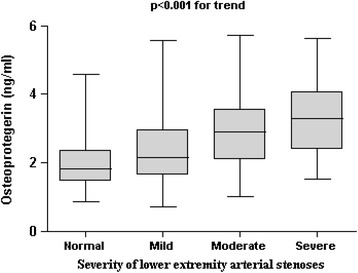
Plasma OPG levels in type 2 diabetes without lower extremity arterial stenosis (Normal) compared with patients with mild-, moderate-, or severe lower extremity arterial stenosis. Boxes represent the 95 % confidence intervals, with the mean superimposed as a horizontal line. Error bars indicate ranges of plasma OPG levels. *p* < 0.001 for trend

The median (inter-quartile range) of OPG concentrations significantly increased for those with normal, mild, moderate and severe was 1.83 (1.41–2.29), 2.02 (1.47–2.59), 2.41 (1.74–3.45), and 2.74 (2.13–3.65) ng/mL, respectively, after adjustment for traditional cardiovascular risk factors such as age, gender, smoking, total cholesterol, HDL-C, CRP, BMI, and SBP (*P* < 0.001 for trend), (Fig. 1). The risk of lower extremity arterial disease was increased (OR = 1.17, 95 % CI 1.09-1.28, *p* < 0.001) with each SD higher level of OPG in patients with type 2 diabetes after adjustment for traditional CVD risk factors (Table 3).

**Table 3:** The risk of lower extremity arterial stenosis associated with 1 SD increase in plasma OPG

Model	Adjustment	lower extremity arterial stenosis [OR, (95 % CI)]	*p* value
Model 1	Unadjusted	1.39 (1.23–1.54)	<0.001
Model 2	Adjusted for age, gender and current smoking	1.35 (1.18–1.49)	<0.001
Model 3	Further adjusted for BMI, waist/hip ratio and HOMA-IR, based on Model 2	1.24 (1.11–1.40)	<0.001
Model 4	Further adjusted for systolic pressure, diastolic pressure, CRP, HbA1c, serum triglycerides, total cholesterol, HDL- and LDL-cholesterol, based on Model 3	1.17 (1.09 –1.28)	<0.001

## Discussion

In this study, we found a strong association between plasma OPG levels and the risk of lower extremity arterial disease in patients with type 2 diabetes. Moreover, this association was independent of traditional CVD risk factors.

OPG was generally considered to be a secreted soluble receptor and was produced by many different tissues and cell types including osteoblasts. Moreover, OPG was produced by cells of the cardiovascular system, including coronary artery smooth muscle cells and endothelial cells, suggesting that alterations of plasma OPG levels may be associated with various atherosclerotic diseases, including endothelial dysfunction, arterial stiffness, aortic atheroma, carotid intima media thickness, and coronary artery disease [15–18]. In line with previous studies [9, 10], our findings also demonstrated that OPG had significant association with lower extremity arterial stenosis, diagnosed by high-frequency color Doppler ultrasonic, in patients with type 2 diabetes. In our study, with lower extremity arterial stenosis patients had significantly elevated plasma OPG concentrations compared with their control counterparts. Our findings, which demonstrated the association between OPG and the severity of lower extremity arterial stenosis, suggest that OPG is a potential biomarker of lower extremity arterial stenosis in type 2 diabetes. Measurement of plasma OPG could be useful for lower extremity arterial stenosis stratification of type 2 diabetes.

The clear mechanism for the vascular effects of OPG is unknown, however, emerging evidence indicates OPG may act as a protective factor for vascular diseases. One hypothesis is that increased serum OPG levels may be a compensatory self-defensive response to the progression of atherosclerosis [8, 19]. It was well-documented that OPG exerts its function through binding and neutralizing the receptor activator for RANK/RANKL system [20]. RANKL can promote an osteogenic differentiation program in vascular smooth muscle cell and stimulate chemokine release, matrix metalloproteinases (MMP)-9 activity, and monocyte/macrophage matrix migration [21]. OPG functions as a soluble decoy receptor for receptor activator of RANK ligand (RANKL or OPG ligand), thereby OPG may serve a protective role in the vascular system. However,some studies indicated OPG may play causal role in atherosclerotic disease. Furthermore, OPG is also a receptor for the cytotoxic ligand TNF-related apoptosis inducing ligand (TRAIL), a potent activator of apoptosis. Recently, OPG has been identified as a survival factor for endothelial cells by blocking TRAIL-induced apoptosis. The regulatory mechanism of OPG on vascular calcification could promote the progression and instability of atherosclerosis [22, 23]. Moreover, a serial of clinical studies have shown that elevated OPG is associated with a higher risk for death and worse clinical outcome in patients with cardiovascular disease [24, 25]. Furthermore, increased OPG level is an independent risk factor for further progression of carotid atherosclerosis in prospective studies [26]. Although we could not determine whether OPG has a protective or promoting role in lower extremity arterial stenosis by the limitation of design of this study, our findings suggested that the OPG might have a role as a biomarker to identifying patient with lower extremity arterial stenosis.

We found that plasma OPG levels were positively correlated with age, duration of diabetes, SBP, CRP. OPG was not associated with BMI, WC, DBP, FPG, HbA1c, uric acid, total cholesterol, triglycerides, LDL-C, HDL-C and adiponectin. The positive relation we found between OPG and age confirmed the results of previous studies in both men and women [27]. This finding suggested that the factors associated with aging may regulate OPG levels. Szulc et al. showed that the relation between OPG and age was more relevant in men by age 40-45 years, at a time in life when age-dependent bone loss begined [28]. The increasing serum OPG level with ageing could be interpreted as a compensatory mechanism counteracting the age progression of bone resorption and atherosclerosis.

To our knowledge, our study is the first one to look at a relationship between OPG and the severity of lower extremity arterial stenosis. However, this study had a number of limitations. First, a prospective study is needed to clarify the causal relationship between OPG and lower extremity arterial stenosis. Second, the relatively small sample size limits the generalizability of our conclusions. Further studies looking at these relations in larger populations such as old and young people should be performed. Third is the fact that circulating concentrations of biomarkers are influenced by a number of factors, mostly unknown, and their usage should be validated in each condition.

## Conclusions

In summary, our data showed that, increased plasma OPG levels were independently associated with the presence and the severity of lower extremity arterial stenosis. This finding suggested that plasma OPG levels were an important determinant of lower extremity arterial atherosclerosis in patients with type 2 diabetes. Although the exact pathophysiologic effect of OPG on lower extremity arterial stenosis is unknown, our findings suggest that plasma OPG is a potential biomarker for lower extremity arterial stenosis. Further studies are warranted to determine the functional role of OPG in the development of atherosclerosis in normal patients.
